# The Many Roles of Galectin-3, a Multifaceted Molecule, in Innate Immune Responses against Pathogens

**DOI:** 10.1155/2017/9247574

**Published:** 2017-05-11

**Authors:** Laura Díaz-Alvarez, Enrique Ortega

**Affiliations:** Departamento de Inmunología, Instituto de Investigaciones Biomédicas, Universidad Nacional Autónoma de México, Ciudad Universitaria, Ciudad de México, Mexico

## Abstract

Galectins are a group of evolutionarily conserved proteins with the ability to bind *β*-galactosides through characteristic carbohydrate-recognition domains (CRD). Galectin-3 is structurally unique among all galectins as it contains a C-terminal CRD linked to an N-terminal protein-binding domain, being the only chimeric galectin. Galectin-3 participates in many functions, both intra- and extracellularly. Among them, a prominent role for Galectin-3 in inflammation has been recognized. Galectin-3 has also been shown to directly bind to pathogens and to have various effects on the functions of the cells of the innate immune system. Thanks to these two properties, Galectin-3 participates in several ways in the innate immune response against invading pathogens. Galectin-3 has been proposed to function not only as a pattern-recognition receptor (PRR) but also as a danger-associated molecular pattern (DAMP). In this review, we analyze the various roles that have been assigned to Galectin-3, both as a PRR and as a DAMP, in the context of immune responses against pathogenic microorganisms.

## 1. Introduction

Galectins are a group of evolutionarily conserved proteins present in vertebrates, invertebrates, and fungi [1]. They possess characteristic carbohydrate-recognition domains (CRD) of about 130 amino acids, through which they have the ability to bind *β*-galactosides (reviewed in [2]). So far, 15 mammalian galectins have been described. They can be structurally classified into three groups: prototype galectins (Galectins 1, 2, 5, 7, 10, 11, 13, 14, and 15) that have a single CRD; tandem galectins (Galectins 4, 6, 8, 9, and 12), with 2 distinct but homologous CRDs; and the chimera-type group, of which Galectin-3 (Gal-3) is the only member, with a C-terminal CRD and a large N-terminal (NT) protein-binding domain (reviewed in [3]).

The human Gal-3 gene (LGALS3) spans 17 Kb and contains 6 exons and 5 introns. It has an open reading frame of 750 bp which translates into a protein of 250 aminoacids [4] with a Mr of approx. 30,000 [5, 6]. As aforementioned, Gal-3 has a unique structure among galectins. The C-terminal half, that is the CRD, is folded into a *β*-sandwich fashion with a tryptophan core and a noncanonical carbohydrate-binding site that mediates interaction with sugars such as N-acetyllactosamine (its preferential ligand), galactomannans, and polymannan [7, 8]. At the other end, the N-terminal region of about 120 residues contains an N-terminal stretch with two potential phosphorylation sites (residues 6 and 12) followed by a region containing several tandem repeats of short amino acid segments (P–G-A-Y-P–G). The glycine- and proline-rich domain is involved in the ability of Gal-3 to oligomerize with other Gal-3 molecules or to establish protein-protein interactions with distinct proteins, like Alix from T cells and CD147 in keratinocytes [7, 9–12].

Gal-3 is widely distributed throughout the body; it can be found in a number of tissues such as the digestive and urogenital tracts, lungs, blood, kidneys, and heart. Gal-3 is highly expressed in myeloid cells (monocytes, macrophages, dendritic cells (DCs), neutrophils, etc.) and fibroblasts, as well as in epithelial and endothelial cells [13–17]. At the cellular level, Gal-3 can be located in the cytoplasm, nucleus, and membranes, and it can also be found extracellularly after being released from cells following different stimuli, like LPS and interferon-*γ*, in both physiological and pathophysiological conditions [18, 19]. Several different functions have been attributed to intracellular Gal-3, including antiapoptotic activity and the regulation of mRNA splicing [20, 21], regulation of the Fc*ε*RI signaling pathway in mast cells [22], and modulation of the activation of RhoA and MLCK during cell invasion in hepatocellular carcinoma [22, 23]. For its part, extracellular Gal-3 (either membrane associated or free) also participates in a wide range of functions, including immunity against pathogens, and in both acute and chronic inflammation. Recent studies have demonstrated that Gal-3 can recognize microbial structures (pathogen-associated molecular patterns), that it has pro-inflammatory properties promoting the infiltration of neutrophils and other immune cells to the infected sites, and that it can also be released as a damage-associated molecular pattern [24]. In this review, we discuss several findings related to the participation of Gal-3 in immunity against pathogens, with special emphasis on its role in innate immunity. The roles of Gal-3 in different physiopathological settings, such as autoimmunity, cancer, and heart failure, have been recently reviewed [25–27].

## 2. Secretion of Galectin-3 in Response to Infection

After its synthesis, Gal-3 is stored in the cytoplasm, where it performs several functions, including some that require its entry into the nucleus. Upon different stimuli such as tissue damage or infection (see below), Gal-3 is either passively released from dying cells or actively secreted by activated cells. Once in the extracellular medium, secreted Gal-3 can act as a pattern-recognition receptor (PRR) and as an activator or modulator of innate immune cells, and it is also considered as a potential damage-associated molecular pattern (DAMP) [28]. Due to the fact that Gal-3 does not bear a typical ER-targeting sequence which would deliver it into a classical secretory pathway, it is secreted through a “leaderless” pathway. Although the precise mechanism by which it exits the cell is not yet fully understood, some details have been elucidated. For instance, a short amino acid sequence close to the N-terminal portion of the protein is known to be required for its extracellular translocation [29].

Gal-3 expression is increased in various epithelial and myeloid cells by microbial and nonmicrobial inflammatory stimuli. Among nonmicrobial stimuli, it is known that Gal-3 is expressed on the surface of human monocytes and its expression level increases upon differentiation to macrophages. Monocytes secrete Gal-3 when stimulated by the calcium ionophore A23187 [30], suggesting that physiological stimuli that induce a rise in cytosolic Ca^2+^ trigger the release of Gal-3.

Different infections induce an increased secretion of Gal-3. Thus, for example, Gal-3 was shown to be upregulated by *Helicobacter pylori* in gastric epithelial cells [31, 32]. Infection with *Neisseria meningitidis* induces a high expression of Gal-3 in spleens of infected mice and in humans with meningococcal infection [33], and both *Candida albicans* and *C. parapsilosis* (the leading causes of neonate candidiasis) upregulate the secretion of Gal-3 in human gingival epithelial cells [34] and neutrophils [35]. Also, an increase in expression of Gal-3 and augmented neutrophil infiltration was detected in the small airways of patients with severe chronic obstructive pulmonary disease [36].

Expression levels of Gal-3 may influence the course of *Mycobacterium leprae* infection. Expression of Gal-3 was studied in two forms of disease due to *M. leprae*: tuberculoid leprosy, which is the mild and self-contained form, and lepromatous leprosy, a more extended and progressive form of the disease. While Gal-3 was nondetectable in self-limited tuberculoid lesions, its expression was highly upregulated in macrophages present in the lesions of patients with lepromatous leprosy. Interestingly, in in vitro assays, it was shown that Gal-3 enhanced the secretion of IL-10 by monocytes stimulated with the TLR2/1 ligand, 19 kD lipopeptide from *M. tuberculosis*, while the secretion of IL-12p40 was unaffected. Moreover, Gal-3 also diminished the ability of monocytes to differentiate into DCs in response to GM-CSF and the ability of monocyte-derived dendritic cells to stimulate proliferation of CD1b-restricted T cells in response to mycobacterial antigens, resulting in a deficient activation of the cell-mediated adaptive immune response which is necessary to control the infection [37]. Thus, in this intracellular infection, high expression of Gal-3 affects the activation of T cells in several ways, favoring the more severe form of the disease.

## 3. Interaction of Galectin-3 with Microbial Ligands

Various lectins serve as PRRs in innate immune responses against pathogens, including soluble molecules such as collectins and ficollins [38], as well as membrane receptors like the members of the C-type lectin family [39]. Likewise, galectins, including Gal-3, have been shown to act as PRRs for the recognition of bacteria, virus, fungi, and parasites (reviewed in [2, 28, 40, 41]).

Galectin-3 has been shown to bind LPS from *Escherichia coli* [42] and *Pseudomonas aeruginosa* [43]. Two independent binding sites for LPS have been proposed on Gal-3 [44]; one of them, inhibitable by lactose, mediates interaction of the CRD of Gal-3 with *β*-galactosides of the polysaccharide moiety of LPS, while the other site, located on the N-terminal domain of Gal-3, interacts with the inner core (lipid A) of LPS [44]. Interaction of *E. coli* LPS with Gal-3, either through its CRD or through its N-terminal domain, results in oligomerization of Gal-3, significantly potentiating the proinflammatory activity of Gal-3 on neutrophils [42]. Conversely, interaction of Gal-3 with bacterial LPS can result in negative regulation of LPS-mediated inflammation, as it was demonstrated in studies in which Gal-3-deficient macrophages showed an elevated production of proinflammatory cytokines upon stimulation with LPS, compared to wild-type (wt) cells. The negative modulation of LPS-induced cytokine production by Gal-3 results in higher susceptibility of Gal-3-deficient mice to LPS-induced endotoxic shock, but the absence of Gal-3 resulted in an increased resistance to infection by *Salmonella* spp. associated with a stronger Th1-type response with higher production of NO and hydrogen peroxide [42, 45]. Gal-3 has also been demonstrated to bind lipooligosaccharides from *Neisseria gonorrhoeae* [46] and mycolic acids from mycobacteria [47]. Interestingly, in contrast to the oligomerization of Gal-3 upon binding to *E. coli* LPS, interaction with mycolic acids was reported to inhibit oligomerization of Gal-3 [47]. Galectin-3 also binds phosphatidylinositol mannosides from mycobacteria and accumulates in phagosomes containing live mycobacteria, mainly on the cytosolic face of the phagosomal membrane [48].

Gal-3 also participates in the recognition of pathogenic fungi, acting conjunctly with Dectin-1 [49]. Dectin-1 is a receptor for *β*-glucans present on the cell wall of fungi. Gal-3 recognizes carbohydrates such as oligomannans on the cell wall of certain fungal species, such as *C. albicans*. It was shown that macrophages must express both Dectin-1 and Gal-3 to be able to secrete TNF-*α* in response to either *Saccharomyces cerevisiae* or the pathogenic *C. albicans* [49]. In another study, it was shown that Gal-3 participates in the recognition of *Candida* spp. by macrophages. Although Gal-3 was not required for recognition and phagocytosis of yeasts, recognition of *β*-1,2-oligomannans (present in *Candida* spp.) allowed macrophages to differentiate between pathogenic (*Candida* spp.) and nonpathogenic (*Saccharomyces* spp.) yeasts [50]. Moreover, it was demonstrated that Gal-3 binding to *C. albicans* that have *β*-1,2-linked oligomannans on the cell surface directly induces the death of *C. albicans* [51]. This antimicrobial activity is restricted to species having *β*-1,2-oligomannans, as Gal-3 did not bind to *S. cerevisiae*, which lacks this type of carbohydrates.

Gal-3 also binds to other microbial pathogens such as the protozoans *Leishmania major* [24] and *Trypanosoma cruzi* [52]. In this last study, Gal-3 was shown to bind to three different proteins of *T. cruzi*, and this binding was inhibited by lactose, suggesting that it was mediated by the CRD of Gal-3. Interaction of Gal-3 with ligands on the surface of pathogens is not always detrimental for the microorganism. For example, Gal-3 can be used by some parasites to favor its attachment to host tissues, like *T. cruzi* that uses Gal-3 to adhere to laminin [52]. Also, Gal-3 is involved in the binding of *T. cruzi* to human coronary artery smooth muscle cells, and silencing Gal-3 gene in these cells dramatically reduces infection, showing that Gal-3 is involved in parasite entry to the cells. The effect of silencing Gal-3 could be reversed by addition of exogenous Gal-3 [53].

## 4. Effects of Galectin-3 on Immune Cells

Apart from directly interacting with pathogens, Gal-3 can affect the course of an infection by virtue of its effects on the cells of the innate immune system such as neutrophils, monocytes/macrophages, and dendritic cells. Circulating blood monocytes and tissue macrophages play important roles in defense against microbial pathogens and in maintaining tissue homeostasis. Tissue-resident macrophages and DCs are located in most organs, especially those in contact with the external environment, such as skin, lungs, and epithelia of the digestive and genital tracts, playing a role as sentinels. They display a vast repertoire of innate receptors to detect pathogens or endogenous molecules released by damaged cells (alarmins) or secreted by activated cells. Upon recognition of these signals, macrophages and DCs become activated and secrete proinflammatory cytokines that trigger an inflammatory response. As already mentioned, Gal-3 is produced and secreted by many cells, including immune cells. Effects of Gal-3 on immune cell functions can be mediated by extracellular Gal-3 binding to membrane receptors on the cell or by intracellular Gal-3 modulating the activity of intracellular proteins. Effects observed by addition of extracellular Gal-3 to cells in in vitro experiments reflect the activity of extracellular Gal-3. The effects of intracellular galectin can be approached either by experimenting with Gal-3-deficient cells from KO mice or by silencing Gal-3 expression in human cells. In the latter cases, however, effects can be produced by intracellular or by extracellular Gal-3, because Gal-3 produced by one cell type can be secreted and act in an autocrine or paracrine way. Therefore, ascertaining if the addition of exogenous Gal-3 to Gal-3 deficient cells can reverse the observed phenomenon is necessary to distinguish intracellular form extracellular effects.

Neutrophils are the most abundant leukocytes in human blood, and they are the first leukocytes to arrive at the sites of tissue injury or presence of pathogens. They are specialized effector cells with the highest phagocytic and microbicidal capacity, but the high destructive potential of some of its products can also cause damage to self-tissues. Exogenously added Gal-3 is able to bind to both naive and primed neutrophils inducing their activation, which is reflected in the shedding of L-selectin and production of IL-8. Activation of neutrophils by Gal-3 depends on both the C-terminal CRD and the N-terminal nonlectin domain [54]. Interestingly, after Gal-3 binding, primed but not naive neutrophils can cleave Gal-3 [54]. In extravasated neutrophils, Gal-3 has been shown to activate the NADPH oxidase [55]. Extravasation can be substituted by in vitro priming and seems to be necessary for the neutrophil to express a receptor for Gal-3 on the cell membrane (presumably mobilized from gelatinase granules) because binding of Gal-3 to neutrophils also increases by priming [55, 56]. In a different study, the possible receptors for Gal-3 were identified as CD66a and CD66b, which are localized in gelatinase and specific granules in resting neutrophils, and are exposed on the cell membrane after priming [57].

In a model of skin infection by *Leishmania major* in mice, Gal-3 deficiency resulted in a decrease in the number of infiltrating neutrophils [58]. It was shown that the injection of exogenous Gal-3 induced neutrophil migration to the site of injection, even though Gal-3 is not a chemoattractant for neutrophils in vitro [59], suggesting that once released from cells, Gal-3 acts as a DAMP to (indirectly) induce neutrophil migration [58]. Gal-3 not only increases neutrophil migration to inflamed tissues but also participates in their removal, a critical process for terminating an inflammatory response. This was first shown in vitro, demonstrating that extracellular Gal-3 acts as an opsonin for the phagocytosis of apoptotic neutrophils by macrophages [60]. Recently, the important role of Gal-3 in neutrophil clearance after an inflammatory response was also demonstrated in vivo in a model of self-resolving peritonitis, where Gal-3-deficient mice showed reduced neutrophil apoptosis and efferocytosis, resulting in an impaired neutrophil clearance [61]. Besides its role in inducing neutrophil migration, Gal-3 might also participate in phagocytosis of *Candida* spp. by neutrophils, because treatment with exogenous Gal-3 results in increased phagocytosis of both *C. albicans* and *C. parapsilosis*, and the exposure of neutrophils to *C. parapsilosis* yeast increased phagocytosis of *C. albicans* and was inhibited by an anti-Gal-3-blocking antibody [35].

Evidence for the role of Gal-3 as a DAMP was obtained from studies of sepsis induced by *Franciscella* spp. infection in mice. Gal-3 was reported to be released in the lungs of mice with lethal infection by *F. novicida*, and it was shown to play an important role in the induction of leukocyte infiltration (mainly neutrophils), release of inflammatory cytokines, vascular injury, and release of inflammatory mediators from neutrophils [62]. In this model, the exacerbated neutrophil influx induced by Gal-3 was at least partly responsible for the damage to the lung tissue, since Gal-3-deficient mice showed reduced cell death and tissue damage, and had better survival rates than wt mice, despite having a similar bacterial burden. It is contrasting that while an elevated release of Gal-3 can produce exacerbated leukocyte infiltration and activation that can result in tissue damage, Gal-3 also has the ability to bind LPS, and therefore, the release of Gal-3 may act as a negative regulator of LPS-induced endotoxic shock [45].

Macrophages are a phenotypically and functionally heterogeneous group of myeloid cells, with high plasticity depending on the local cytokine milieu. In tissues, macrophages respond to cytokines and other signals with the acquisition of distinct functional phenotypes. In response to TLR ligands and IFN-*γ*, macrophages undergo classical M1 activation, whereas, after stimulation with IL-4 or IL-10, they attain alternative M2 activation phenotypes. The M1 phenotype is characterized by the expression of high levels of proinflammatory cytokines, high production of reactive nitrogen and oxygen intermediates, promotion of Th1-type responses, and strong microbicidal and tumoricidal activity, while M2 macrophages are involved in parasite containment and promotion of tissue remodeling and tumor progression and have immunoregulatory functions. Gal-3 was found to regulate macrophage polarization to the M2 phenotype, as macrophages from Gal-3-deficient mice showed impaired polarization in response to IL-4 [63]. Since macrophage polarization by IL-4 was shown to upregulate Gal-3 expression and secretion and IL-4-induced alternative activation is blocked by a specific inhibitor of carbohydrate binding to Gal-3, the results suggest that IL-4 triggers secretion of Gal-3 that, in a positive feedback loop, acts extracellularly to promote alternative activation of macrophages [63].

In human monocytes, exogenously added Gal-3 induces production of superoxide [30], and as above-mentioned, Gal-3 also inhibits their differentiation into DCs in vitro [37]. In the cells of the microglia (mononuclear phagocytes of the central nervous system), secreted Gal-3 acts as an autocrine ligand for TLR4 and induces TLR4-mediated activation [64]. Gal-3 was also shown to regulate activation of macrophages in a model of dextran-induced colitis in mice [65]. It was previously shown that colitis in this model is mediated by the NLRP3 inflammasome [66]. Gal-3 deficiency or its inhibition with GM-CT-01 (a galactomannan, [67]) resulted in a significant attenuation of DSS-induced colitis. This was associated with a significantly lower production of TNF and IL-1*β* and reduced activation of the NLRP3 inflammasome in peritoneal macrophages from Gal-3-deficient mice treated in vitro with LPS or DSS [65].

Dendritic cells capture antigens on peripheral tissues, secrete proinflammatory cytokines, and migrate into draining lymph nodes where they are essential for presenting antigen and secreting cytokines for the activation of T cells. Furthermore, by integrating signals derived from the pathogen and the microenvironment and translating these into patterns of cytokines secreted, DCs play a central role in determining the Th1/Th2/Th17 polarization of the adaptive immune response. Thus, DCs are pivotal for linking innate and adaptive immunity.

There are several ways in which Gal-3 affects the functions of DCs. It was reported that infection with *T. cruzi* modulates (both in vivo and in vitro) the expression of Gal-3 in splenic DCs from infected mice and in the immortalized dendritic cell line D2SC-1 [68]. Because infected D2SC-1 cells showed reduced migration on Gal-3-coated surfaces, it can be speculated that secretion of Gal-3 by infected cells will result in an inhibition of their migration to secondary lymphoid organs, as secreted Gal-3 would bind to extracellular matrix proteins, and by interacting with DCs would inhibit their migration and therefore T cell activation. In a model of a different parasitic infection, it was found that oral infection of mice with *Toxoplasma gondii* induced a high expression of Gal-3 in leukocytes infiltrating the intestine, lungs, liver, and brain. Compared to infected wt mice, infected Gal-3-deficient mice showed a reduced inflammatory response in the intestine, liver, and brain and a higher parasite burden in the brain. Dendritic cells from Gal-3-deficient mice showed a higher production of IL-12, and this was reflected in a stronger Th1-polarized response in Gal-3-deficient mice. In this model, while the proinflammatory activity is most probably mediated by extracellular Gal-3, intracellular Gal-3 modulates cytokine secretion by DCs and, in this way, affects the adaptive immune response [69]. A further example of the modulation of an adaptive immune response by Gal-3 through its effects on DCs was evident in a model of histoplasmosis in mice. It is known that in this model, Th1 and Th17 responses contribute to eliminate the infection. It was reported that DCs from infected Gal-3-deficient mice produced significantly higher levels of cytokines of the IL-23/IL-17 axis and lower levels of IL-12 and IFN-*γ* than cells from wt mice [70]. Consequently, Gal-3-deficient mice had an increased number of Th17 cells and a reduction in Th1 cells and a lower fungal burden. These contrasting effects of Gal-3 deficiency on the induction of a Th1 or Th17 response in these two models (toxoplasmosis and histoplasmosis) highlight the complexity of the mechanisms regulating immune responses and warn against simplistic generalizations derived from studies of deficiency of a single molecule in a single experimental setting. Gal-3 also modulates cytokine secretion in human dendritic cells. Gal-3 siRNA downregulates expression of proinflammatory IL-6, IL-1*β*, and IL-23 and upregulates expression of IL-10 and IL-12p35 in human monocyte-derived dendritic cells (MoDCs) stimulated with TLR ligands (LPS and R484). The change in cytokine secretion profile influenced the production of cytokines by T CD4 cells stimulated in the presence of Gal-3 siRNA-treated MoDCs. Since neutralizing antibodies against Gal-3 or exogenous recombinant Gal-3 did not reverse the effects of Gal-3 siRNA, the data points to the participation of intracellular Gal-3 in the regulation of cytokine secretion in DCs [71].

## 5. Galectin-3 in Infection by *Helicobacter pylori*

Infection of gastric epithelial cells by *H. pylori* induces secretion of Gal-3 into the superficial mucus layer, and this is important for trapping bacteria in the mucus layer preventing attachment to and infection of epithelial cells. This is reflected in the fact that in Gal-3-deficient mice, *H. pylori* infiltrated deeply into the gastric tissue at 2 weeks postinfection, and bacterial loads were higher at 2 weeks and 6 months postinoculation [72]. Secretion of Gal-3 induced by infection of gastric epithelial cells by *H. pylori* [31] occurs by a mechanism postulated to involve binding of the O-antigen side chain of *H. pylori* LPS to membrane Gal-3; coincidently, it was recently shown that extracellular recombinant Gal-3 is able to inhibit adhesion of *H. pylori* to the gastric epithelium [73]. Gal-3 also influenced killing of *H. pylori*, since, although peritoneal macrophages from both wt and Gal-3-deficient mice ingested *H. pylori* efficiently, Gal-3-deficient cells were inefficient in killing engulfed *H. pylori* in vitro. Moreover, extracellular recombinant Gal-3 was also shown to have a potent bactericidal action on *H. pylori* [72]. The effect is inhibited by lactose, suggesting that the CRD of Gal-3 is involved in the elimination of *H. pylori* by the macrophages.

A possible relationship between *H. pylori*, Gal-3, and gastric cancer was revealed in studies showing upregulation of Gal-3 in cancer gastric cells upon infection by *H. pylori*, resulting in both elevated Gal-3 secretion and higher levels of cytoplasmic Gal-3. In these cells, intracellular Gal-3 was shown to be proproliferative and to inhibit apoptosis, resulting in the extended cell survival characteristic of carcinogenesis [74]. In a different study, elevated intracellular Gal-3 was postulated to be involved in the resistance to growth arrest produced by IFN-*γ* in a hyperproliferative gastric cancer cell line [75].

## 6. Galectin-3 in Infections by *Neisseria meningitidis*

Infection with *N. meningitidis* induces elevated expression of Gal-3 in the spleens of infected mice and humans with meningococcal infection. It has been demonstrated that Gal-3 binds to *N. meningitidis* and that this interaction requires intact lipopolysaccharide molecules on the surface of the bacterium. Recombinant Gal-3 added exogenously increased adhesion of *N. meningitidis* to THP-1 monocytes and to human primary monocytes, although it did not result in increased internalization of bacteria [33]. The possible relevance of Gal-3 in infections by *N. meningitidis* in vivo was investigated in Gal-3-deficient mice. Surprisingly, Gal-3-deficient mice had significantly lower levels of bacteraemia compared with wt mice [33], suggesting that Gal-3 enhances survival of the bacteria. Although the mechanisms for this effect are not clear, the authors propose that the increased attachment of bacteria to phagocytes protects bacteria from phagocytosis by neighboring cells, affects the activity of the phagocyte, or facilitates dissemination.

The interaction of Gal-3 with *N. meningitidis* and the role of Gal-3 in the entry of bacteria to eukaryotic cells were studied in N2a cells (which do not express endogenous Gal-3) transfected with Gal-3-YFP [76] or by silencing Gal-3 expression in human cells. This study found that besides LPS-dependent binding, Gal-3 also binds meningococci through a LPS-independent interaction. Gal-3 is able to form heterodimers with 37LRP, which is a precursor of the laminin receptor LAMR1. Gal-3-37LRP heterodimers were proposed to mediate increased bacterial attachment by binding to PilE, strengthening the initial contact, inducing pilus retraction, and allowing the subunit PilQ to bind to both Gal-3 and 37LRP on the cell surface, contributing to cell invasion [76].

## 7. Galectin-3 in Infection by *Streptococcus pneumoniae*

Galectin-3 production has also been reported in infection of the airways, and in some cases, participation of Gal-3 can be detrimental to the host. Thus, it was revealed that binding of Gal-3 (whose release is induced during infection by influenza A virus) to the surface of epithelial cells modulates the expression of RIG-1 and SOCS-1 and the activation of ERK, AKT, and JAK/STAT1 signaling pathways, resulting in a deregulated release of proinflammatory cytokines [77]. Moreover, Gal-3 released by the airway epithelium during infection with influenza A virus does not only deregulates cytokine production but also facilitates the adhesion of *Streptococcus pneumoniae* to the epithelium, contributing to the increased susceptibility of influenza patients to infections by *S. pneumoniae* [78]. However, in the absence of infection by influenza virus, the production of Gal-3 during infection by *S. pneumoniae* is significant for controlling the infection. Gal-3 also facilitates the recruitment of neutrophils to the lungs [79]. This was confirmed using Gal-3-deficient mice, by showing that in these mice a lower number of neutrophils were recruited during infection. Interestingly, Gal-3 seems to participate specifically in migration that is not dependent on *β*-2 integrins, as neutrophil recruitment during infection by *E. coli* (known to depend on *β*-2 integrins) was not affected [80]. The role of Gal-3 during lung infection by *S. pneumoniae* goes beyond recruitment of neutrophils, as was shown in Gal-3-deficient mice, which develop a more severe pneumonia with increased bacteraemia and tissue damage. Investigation into the mechanisms involved showed that in this infection, Gal-3 acts directly as a neutrophil-activating agent potentiating the effect of fMLP, increasing neutrophil phagocytosis of bacteria, and delaying neutrophil apoptosis, apart from being bacteriostatic against *S. pneumoniae* in vitro [81].

## 8. Concluding Remarks

**Figure 1:**
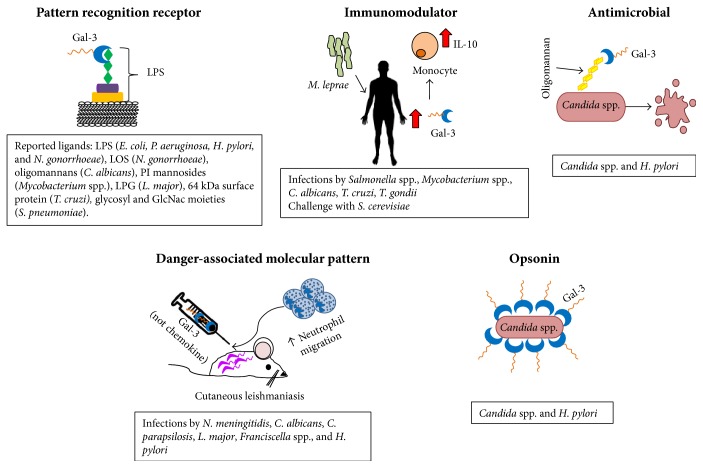
Different activities of Galectin-3 in the immune response against pathogens. Gal-3 = Galectin 3, LPS = lipopolysaccharide, LOS = lipooligosaccharides, PI mannosides = phosphatidylinositol mannosides, LPG = lipophosphoglycan, GlcNac = N-acetylglucosamine, *E. coli* = *Escherichia coli*, *P. aeruginosa* = *Pseudomonas aeruginosa*, *H. pylori* = *Helicobacter pylori*, *N. gonorrhoeae* = *Neisseria gonorrhoeae*, *C. albicans* = *Candida albicans*, *L. major* = *Leishmania major*, *T. cruzi* = *Trypanosoma cruzi*, *S. pneumoniae* = *Streptococcus pneumoniae*, *M. leprae* = *Mycobacterium leprae*, *S. cerevisiae* = *Saccharomyces cerevisiae*, *N. meningitidis* = *Neisseria meningitidis*, *C. parapsilosis* = *Candida parapsilosis*, *T. gondii* = *Toxoplasma gondii*.

Galectin-3, the only chimeric galectin, is a multifunctional molecule with proinflammatory activity that can be found either as a soluble (extracellular or cytoplasmic) or membrane-bound molecule. Gal-3 participates in many different functions, including the regulation of cell growth, differentiation and apoptosis, regulation of RNA splicing, and cell migration (reviewed in [82]). Gal-3 also contributes in several ways to the innate immune response (Figure 1()); it recognizes structures on pathogens and thus is considered as a PRR, and has also been shown to modulate the activity of the cells of the innate immune system in several ways: influencing their migration to the sites of inflammation, increasing their effector functions (like activation of NAPDH oxidase), modulating the production of regulatory cytokines, and even regulating their apoptosis. As can be appreciated from the work summarized above, the roles that Gal-3 can have in the course of an infection are quite diverse and will depend on many factors, such as the localization of Gal-3 itself (extracellular, membrane bound, or cytoplasmic), its degree of oligomerization, and the proteins or cells that it could interact with. Thus, despite the great amount of data that has been generated on the actions of Gal-3, several questions still have to be answered before a clearer picture of the mechanisms and regulation of Gal-3 activities emerge.

A critical point to consider when analyzing the activities of Gal-3 (or any other galectin) is their broad ligand specificity. Galectins bind *β*-glactosides such as lactosamine (Gal*β*1-4GlcNAc), but different modifications/substitutions, multiplicity of this basic unit, or the oligosaccharide context in which this basic structure is present, are important for the affinity of binding [83]. As potential saccharide ligands for Gal-3 are present on many different glycoconjugates, defining the relevant ligand(s) of Gal-3 for each of its particular activities is a complicated issue that goes well beyond demonstrating an interaction in controlled binding experiments in vitro. Similarly, as already mentioned, the non-CRD N-terminal domain can mediate interactions of Gal-3 with distinct molecules, and this domain has also been shown to modulate the binding affinity of the CRD for different saccharides [83], so that definition of the contribution of each domain to the interaction of Gal-3 with its ligands is important. Glycosidic structures bound to glycoconjugates can be modified depending on several parameters, both during the glycosylation of newly synthesized proteins in the endoplasmic reticulum, and by posttranslational modifications. Glycosylation isoforms of a protein could be differentially recognized by Gal-3, and this may be important for regulating some functions of Gal-3. An interesting area for future research is the possible regulation of the recognition by Gal-3, by modifications of the glycan structure of the glycoconjugate. Moreover, it should be kept in mind that Gal-3 can also be posttranslationally modified, and the impact of such modifications on its activity has not been studied in detail.

How does binding of Gal-3 modulates the activity of its ligand is another area in which much is still to be defined. It is likely that in some scenarios, aggregation of glycoproteins by Gal-3 is the critical step, but this is not necessarily so in all instances. Additionally, oligomerization of Gal-3 might promote the formation not only of the homo-oligomers of its ligands but also of the aggregates of different glycoconjugates, and this could have important functional consequences.

Much work has been conducted comparing infections in Gal-3-deficient and wt mice. For a protein with so many different activities at different cellular levels (nuclear, cytoplasmic, membrane, or extracellular), it is difficult to elucidate its functions from comparisons of mice with complete absence of the protein with wt mice. Studies in which deficiency of Gal-3 is specific for a cell type and even for a particular developmental or maturation state are much needed.

Because of its multiple activities and binding of glycans on microbial and immune cells, Gal-3 might have a role in many more processes than those already described. An area worth exploring is the interaction of Gal-3 with the microbiota [84]. Gal-3 is known to be expressed by epithelial cells of the digestive tract, and as aforementioned, interaction of Gal-3 with LPS can modulate proinflammatory cytokine production, and the potential role of Gal-3 in maintaining tolerance toward microbiota in the intestinal tract is an interesting possibility. Of course, defining the relevant ligand(s) and the mechanisms involved is essential for the potential utilization of Gal-3 to sustain a healthy microbiota. The complexity of the myriad of activities described for Gal-3, the difficulty in determining the relevant ligands, and the fact that some of the activities reported might seem contradictory make the precise dissection of the biological role of Gal-3 a great challenge. However, it is a challenge that holds the promise of expanding our understanding of the biology of a family of evolutionarily conserved proteins (galectins) and of its potential therapeutic applications as modulators of inflammation and immune responses.

## References

[1] Vasta G. R., Quesenberry M., Ahmed H., O'Leary N. (1999). C-type lectins and galectins mediate innate and adaptive immune functions: their roles in the complement activation pathway. *Developmental and Comparative Immunology*.

[2] Vasta G. R. (2012). Galectins as pattern recognition receptors: structure, function, and evolution. *Advances in Experimental Medicine and Biology*.

[3] Nio-Kobayashi J. (2017). Tissue- and cell-specific localization of galectins, beta-galactose-binding animal lectins, and their potential functions in health and disease. *Anatomical Science International*.

[4] Kadrofske M. M., Openo K. P., Wang J. L. (1998). The human LGALS3 (galectin-3) gene: determination of the gene structure and functional characterization of the promoter. *Archives of Biochemistry and Biophysics*.

[5] Raz A., Carmi P., Raz T., Hogan V., Mohamed A., Wolman S. R. (1991). Molecular cloning and chromosomal mapping of a human galactoside-binding protein. *Cancer Research*.

[6] Oda Y., Leffler H., Sakakura Y., Kasai K., Barondes S. H. (1991). Human breast carcinoma cDNA encoding a galactoside-binding lectin homologous to mouseMac-2 antigen. *Gene*.

[7] Miller M. C., Ippel H., Suylen D. (2016). Binding of polysaccharides to human galectin-3 at a noncanonical site in its carbohydrate recognition domain. *Glycobiology*.

[8] Agrwal N., Sun Q., Wang J. L., Wang S. Y. (1993). Carbohydrate-binding protein 35. I. Properties of the recombinant polypeptide and the individuality of the domains. *The Journal of Biological Chemistry*.

[9] Wang S. F., Tsao C. H., Lin Y. T. (2014). Galectin-3 promotes HIV-1 budding via association with Alix and Gag p6. *Glycobiology*.

[10] Mauris J., Woodward A. M., Cao Z., Panjwani N., Argüeso P. (2014). Molecular basis for MMP9 induction and disruption of epithelial cell-cell contacts by galectin-3. *Journal of Cell Science*.

[11] Cherayil B. J., Chaitovitz S., Wong C., Pillai S. (1990). Molecular cloning of a human macrophage lectin specific for galactose. *Proceedings of the National Academy of Sciences of the United States of America*.

[12] Ippel H., Miller M. C., Vèrtesy S. (2016). Intra- and intermolecular interactions of human galectin-3: assessment by full-assignment-based NMR. *Glycobiology*.

[13] Nio-Kobayashi J., Boswell L., Amano M., Iwanaga T., Duncan W. C. (2014). The loss of luteal progesterone production in women is associated with a galectin switch via α2,6-sialylation of glycoconjugates. *The Journal of Clinical Endocrinology and Metabolism*.

[14] Nishi Y., Sano H., Kawashima T. (2007). Role of galectin-3 in human pulmonary fibrosis. *Allergology International*.

[15] Yan X. J., Yu G. F., Jie Y. Q., Fan X. F., Huang Q., Dai W. M. (2016). Role of galectin-3 in plasma as a predictive biomarker of outcome after acute intracerebral hemorrhage. *Journal of the Neurological Sciences*.

[16] Saccon F., Gatto M., Ghirardello A., Iaccarino L., Punzi L., Doria A. (2017). Role of galectin-3 in autoimmune and non-autoimmune nephropathies. *Autoimmunity Reviews*.

[17] Chen J. J., Hao W. R., Chang K. C., Liu J. C. (2016). LBOS 02-03 The infiltrating macrophage-secreted galectin-3 plays an essential role in cardiac fibrosis and diastolic function in murine pressure-overload model. *Journal of Hypertension*.

[18] Fritsch K., Mernberger M., Nist A., Stiewe T., Brehm A., Jacob R. (2016). Galectin-3 interacts with components of the nuclear ribonucleoprotein complex. *BMC Cancer*.

[19] Kang H. G., Kim D. H., Kim S. J. (2016). Galectin-3 supports stemness in ovarian cancer stem cells by activation of the Notch1 intracellular domain. *Oncotarget*.

[20] Haudek K. C., Spronk K. J., Voss P. G., Patterson R. J., Wang J. L., Arnoys E. J. (2010). Dynamics of galectin-3 in the nucleus and cytoplasm. *Biochimica et Biophysica Acta*.

[21] Dagher S. F., Wang J. L., Patterson R. J. (1995). Identification of galectin-3 as a factor in pre-mRNA splicing. *Proceedings of the National Academy of Sciences of the United States of America*.

[22] Bambouskova M., Polakovicova I., Halova I. (2016). New regulatory roles of Galectin-3 in high-affinity IgE receptor signaling. *Molecular and Cellular Biology*.

[23] Serizawa N., Tian J., Fukada H. (2015). Galectin 3 regulates HCC cell invasion by RhoA and MLCK activation. *Laboratory Investigation*.

[24] Sato S., Bhaumik P., St-Pierre G., Pelletier I. (2014). Role of galectin-3 in the initial control of Leishmania infection. *Critical Reviews in Immunology*.

[25] de Oliveira F. L., Gatto M., Bassi N. (2015). Galectin-3 in autoimmunity and autoimmune diseases. *Experimental Biology and Medicine (Maywood, N.J.)*.

[26] Wang L., Guo X. L. (2016). Molecular regulation of galectin-3 expression and therapeutic implication in cancer progression. *Biomedicine & Pharmacotherapy*.

[27] Hrynchyshyn N., Jourdain P., Desnos M., Diebold B., Funck F. (2013). Galectin-3: a new biomarker for the diagnosis, analysis and prognosis of acute and chronic heart failure. *Archives of Cardiovascular Diseases*.

[28] Sato S., St-Pierre C., Bhaumik P., Nieminen J. (2009). Galectins in innate immunity: dual functions of host soluble beta-galactoside-binding lectins as damage-associated molecular patterns (DAMPs) and as receptors for pathogen-associated molecular patterns (PAMPs). *Immunological Reviews*.

[29] Mehul B., Hughes R. C. (1997). Plasma membrane targetting, vesicular budding and release of galectin 3 from the cytoplasm of mammalian cells during secretion. *Journal of Cell Science*.

[30] Liu F. T., Hsu D. K., Zuberi R. I., Kuwabara I., Chi E. Y., Henderson W. R. (1995). Expression and function of galectin-3, a beta-galactoside-binding lectin, in human monocytes and macrophages. *The American Journal of Pathology*.

[31] Fowler M., Thomas R. J., Atherton J., Roberts I. S., High N. J. (2006). Galectin-3 binds to Helicobacter pylori O-antigen: it is upregulated and rapidly secreted by gastric epithelial cells in response to H. pylori adhesion. *Cellular Microbiology*.

[32] Lim J. W., Kim H., Kim K. H. (2003). Cell adhesion-related gene expression by Helicobacter pylori in gastric epithelial AGS cells. *The International Journal of Biochemistry & Cell Biology*.

[33] Quattroni P., Li Y., Lucchesi D. (2012). Galectin-3 binds Neisseria meningitidis and increases interaction with phagocytic cells. *Cellular Microbiology*.

[34] Tamai R., Kiyoura Y. (2014). *Candida albicans* and *Candida parapsilosis* rapidly up-regulate galectin-3 secretion by human gingival epithelial cells. *Mycopathologia*.

[35] Linden J. R., Kunkel D., Laforce-Nesbitt S. S., Bliss J. M. (2013). The role of galectin-3 in phagocytosis of *Candida albicans* and *Candida parapsilosis* by human neutrophils. *Cellular Microbiology*.

[36] Pilette C., Colinet B., Kiss R. (2007). Increased galectin-3 expression and intra-epithelial neutrophils in small airways in severe COPD. *The European Respiratory Journal*.

[37] Chung A. W., Sieling P. A., Schenk M. (2013). Galectin-3 regulates the innate immune response of human monocytes. *The Journal of Infectious Diseases*.

[38] Foo S. S., Reading P. C., Jaillon S., Mantovani A., Mahalingam S. (2015). Pentraxins and collectins: friend or foe during pathogen invasion?. *Trends in Microbiology*.

[39] Sancho D., Reis e Sousa, C. (2012). Signaling by myeloid C-type lectin receptors in immunity and homeostasis. *Annual Review of Immunology*.

[40] Chen H. Y., Weng I. C., Hong M. H., Liu F. T. (2014). Galectins as bacterial sensors in the host innate response. *Current Opinion in Microbiology*.

[41] Vasta G. R. (2009). Roles of galectins in infection. *Nature Reviews. Microbiology*.

[42] Fermino M. L., Polli C. D., Toledo K. A. (2011). LPS-induced galectin-3 oligomerization results in enhancement of neutrophil activation. *PloS One*.

[43] Gupta S. K., Masinick S., Garrett M., Hazlett L. D. (1997). Pseudomonas aeruginosa lipopolysaccharide binds galectin-3 and other human corneal epithelial proteins. *Infection and Immunity*.

[44] Mey A., Leffler H., Hmama Z., Normier G., Revillard J. P. (1996). The animal lectin galectin-3 interacts with bacterial lipopolysaccharides via two independent sites. *Journal of Immunology*.

[45] Li Y., Komai-Koma M., Gilchrist D. S. (2008). Galectin-3 is a negative regulator of lipopolysaccharide-mediated inflammation. *Journal of Immunology*.

[46] John C. M., Jarvis G. A., Swanson K. V. (2002). Galectin-3 binds lactosaminylated lipooligosaccharides from Neisseria gonorrhoeae and is selectively expressed by mucosal epithelial cells that are infected. *Cellular Microbiology*.

[47] Barboni E., Coade S., Fiori A. (2005). The binding of mycolic acids to galectin-3: a novel interaction between a host soluble lectin and trafficking mycobacterial lipids?. *FEBS Letters*.

[48] Beatty W. L., Rhoades E. R., Hsu D. K., Liu F. T., Russell D. G. (2002). Association of a macrophage galactoside-binding protein with Mycobacterium-containing phagosomes. *Cellular Microbiology*.

[49] Esteban A., Popp M. W., Vyas V. K., Strijbis K., Ploegh H. L., Fink G. R. (2011). Fungal recognition is mediated by the association of dectin-1 and galectin-3 in macrophages. *Proceedings of the National Academy of Sciences of the United States of America*.

[50] Jouault T., El Abed-El Behi M., Martinez-Esparza M. (2006). Specific recognition of *Candida albicans* by macrophages requires galectin-3 to discriminate *Saccharomyces cerevisiae* and needs association with TLR2 for signaling. *Journal of Immunology*.

[51] Kohatsu L., Hsu D. K., Jegalian A. G., Liu F. T., Baum L. G. (2006). Galectin-3 induces death of Candida species expressing specific beta-1,2-linked mannans. *Journal of Immunology*.

[52] Moody T. N., Ochieng J., Villalta F. (2000). Novel mechanism that Trypanosoma cruzi uses to adhere to the extracellular matrix mediated by human galectin-3. *FEBS Letters*.

[53] Kleshchenko Y. Y., Moody T. N., Furtak V. A., Ochieng J., Lima M. F., Villalta F. (2004). Human galectin-3 promotes Trypanosoma cruzi adhesion to human coronary artery smooth muscle cells. *Infection and Immunity*.

[54] Nieminen J., St-Pierre C., Sato S. (2005). Galectin-3 interacts with naive and primed neutrophils, inducing innate immune responses. *Journal of Leukocyte Biology*.

[55] Karlsson A., Follin P., Leffler H., Dahlgren C. (1998). Galectin-3 activates the NADPH-oxidase in exudated but not peripheral blood neutrophils. *Blood*.

[56] Almkvist J., Faldt J., Dahlgren C., Leffler H., Karlsson A. (2001). Lipopolysaccharide-induced gelatinase granule mobilization primes neutrophils for activation by galectin-3 and formylmethionyl-Leu-Phe. *Infection and Immunity*.

[57] Feuk-Lagerstedt E., Jordan E. T., Leffler H., Dahlgren C., Karlsson A. (1999). Identification of CD66a and CD66b as the major galectin-3 receptor candidates in human neutrophils. *Journal of Immunology*.

[58] Bhaumik P., St-Pierre G., Milot V., St-Pierre C., Sato S. (2013). Galectin-3 facilitates neutrophil recruitment as an innate immune response to a parasitic protozoa cutaneous infection. *Journal of Immunology*.

[59] Baseras B., Gaida M. M., Kahle N. (2012). Galectin-3 inhibits the chemotaxis of human polymorphonuclear neutrophils in vitro. *Immunobiology*.

[60] Karlsson A., Christenson K., Matlak M. (2009). Galectin-3 functions as an opsonin and enhances the macrophage clearance of apoptotic neutrophils. *Glycobiology*.

[61] Wright R. D., Souza P. R., Flak M. B., Thedchanamoorthy P., Norling L. V., Cooper D. (2017). Galectin-3-null mice display defective neutrophil clearance during acute inflammation. *Journal of Leukocyte Biology*.

[62] Mishra B. B., Li Q., Steichen A. L. (2013). Galectin-3 functions as an alarmin: pathogenic role for sepsis development in murine respiratory tularemia. *PloS One*.

[63] MacKinnon A. C., Farnworth S. L., Hodkinson P. S. (2008). Regulation of alternative macrophage activation by galectin-3. *Journal of Immunology*.

[64] Burguillos M. A., Svensson M., Schulte T. (2015). Microglia-secreted Galectin-3 acts as a Toll-like receptor 4 ligand and contributes to microglial activation. *Cell Reports*.

[65] Simovic Markovic B., Nikolic A., Gazdic M. (2016). Galectin-3 plays an important pro-inflammatory role in the induction phase of acute colitis by promoting activation of NLRP3 inflammasome and production of IL-1beta in macrophages. *Journal of Crohn's & Colitis*.

[66] Bauer C., Duewell P., Mayer C. (2010). Colitis induced in mice with dextran sulfate sodium (DSS) is mediated by the NLRP3 inflammasome. *Gut*.

[67] Traber P. G., Chou H., Zomer E. (2013). Regression of fibrosis and reversal of cirrhosis in rats by galectin inhibitors in thioacetamide-induced liver disease. *PloS One*.

[68] Vray B., Camby I., Vercruysse V. (2004). Up-regulation of galectin-3 and its ligands by Trypanosoma cruzi infection with modulation of adhesion and migration of murine dendritic cells. *Glycobiology*.

[69] Bernardes E. S., Silva N. M., Ruas L. P. (2006). Toxoplasma gondii infection reveals a novel regulatory role for galectin-3 in the interface of innate and adaptive immunity. *The American Journal of Pathology*.

[70] Wu S. Y., Yu J. S., Liu F. T., Miaw S. C., Wu-Hsieh B. A. (2013). Galectin-3 negatively regulates dendritic cell production of IL-23/IL-17-axis cytokines in infection by Histoplasma capsulatum. *Journal of Immunology*.

[71] Chen S. S., Sun L. W., Brickner H., Sun P. Q. (2015). Downregulating galectin-3 inhibits proinflammatory cytokine production by human monocyte-derived dendritic cells via RNA interference. *Cellular Immunology*.

[72] Park A. M., Hagiwara S., Hsu D. K., Liu F. T., Yoshie O. (2016). Galectin-3 plays an important role in innate immunity to gastric infection by Helicobacter pylori. *Infection and Immunity*.

[73] Subhash V. V., Ling S. S., Ho B. (2016). Extracellular galectin-3 counteracts adhesion and exhibits chemoattraction in Helicobacter pylori-infected gastric cancer cells. *Microbiology*.

[74] Subhash V. V., Ho B. (2016). Galectin 3 acts as an enhancer of survival responses in H. pylori-infected gastric cancer cells. *Cell Biology and Toxicology*.

[75] Tseng P. C., Chen C. L., Shan Y. S., Lin C. F. (2016). An increase in galectin-3 causes cellular unresponsiveness to IFN-gamma-induced signal transduction and growth inhibition in gastric cancer cells. *Oncotarget*.

[76] Alqahtani F., Mahdavi J., Wheldon L. M. (2014). Deciphering the complex three-way interaction between the non-integrin laminin receptor, galectin-3 and Neisseria meningitidis. *Open Biol*.

[77] Nita-Lazar M., Banerjee A., Feng C., Vasta G. R. (2015). Galectins regulate the inflammatory response in airway epithelial cells exposed to microbial neuraminidase by modulating the expression of SOCS1 and RIG1. *Molecular Immunology*.

[78] Nita-Lazar M., Banerjee A., Feng C. (2015). Desialylation of airway epithelial cells during influenza virus infection enhances pneumococcal adhesion via galectin binding. *Molecular Immunology*.

[79] Sato S., Ouellet N., Pelletier I., Simard M., Rancourt A., Bergeron M. G. (2002). Role of galectin-3 as an adhesion molecule for neutrophil extravasation during streptococcal pneumonia. *Journal of Immunology*.

[80] Nieminen J., St-Pierre C., Bhaumik P., Poirier F., Sato S. (2008). Role of galectin-3 in leukocyte recruitment in a murine model of lung infection by Streptococcus pneumoniae. *Journal of Immunology*.

[81] Farnworth S. L., Henderson N. C., Mackinnon A. C. (2008). Galectin-3 reduces the severity of pneumococcal pneumonia by augmenting neutrophil function. *The American Journal of Pathology*.

[82] Dumic J., Dabelic S., Flogel M. (2006). Galectin-3: an open-ended story. *Biochimica et Biophysica Acta*.

[83] Hirabayashi J., Hashidate T., Arata Y. (2002). Oligosaccharide specificity of galectins: a search by frontal affinity chromatography. *Biochimica et Biophysica Acta*.

[84] de Kivit S., Kraneveld A. D., Garssen J., Willemsen L. E. (2011). Glycan recognition at the interface of the intestinal immune system: target for immune modulation via dietary components. *European Journal of Pharmacology*.

